# SEOM clinical guideline on unknown primary cancer (2017)

**DOI:** 10.1007/s12094-017-1807-y

**Published:** 2017-12-11

**Authors:** F. Losa, G. Soler, A. Casado, A. Estival, I. Fernández, S. Giménez, F. Longo, R. Pazo-Cid, J. Salgado, M. Á. Seguí

**Affiliations:** 1 Hospital de Sant Joan Despí Moisés Broggi, Sant Joan Despí, Barcelona Spain; 2 Hospital Durán i Reynals (ICO-L’Hospitalet), Barcelona, Spain; 3 Hospital Universitario Clínico San Carlos, Madrid, Spain; 4 Hospital Universitari Germans Trias i Pujol, Barcelona, Spain; 5 Hospital Alvaro Cunqueiro-Complejo Hospitalario Universitario, Vigo, Spain; 6 Hospital Universitari I Politècnic la Fe, Valencia, Spain; 7 Hospital Universitario Ramón y Cajal, Madrid, Spain; 8 Hospital Universitario Miguel Servet, Zaragoza, Spain; 9 Complejo Hospitalario de Navarra, Pamplona, Spain; 10 Parc Taulí Sabadell, Hospital Universitari, Sabadell, Barcelona Spain

**Keywords:** Cancer, Unknown primary site, Chemotherapy, Diagnosis and treatment

## Abstract

Cancer of unknown primary site is a histologically confirmed cancer that manifests in advanced stage, with no identifiable primary site following standard diagnostic procedures. Patients are initially categorized based on the findings of the initial biopsy: adenocarcinoma, squamous-cell carcinoma, neuroendocrine carcinoma, and poorly differentiated carcinoma. Appropriate patient management requires understanding several clinical and pathological features that aid in identifying several subsets of patients with more responsive tumors.

## Introduction

Cancer of unknown primary site (CUP) is defined as a group of metastatic tumors for which a standardized diagnostic work-up fails to identify the site of origin at the time of diagnosis.

Currently, CUP accounts for 3–5% of all tumors and is among the 10 most frequent tumors in developed countries. It affects both genders equally and the average age at diagnosis is 60 [1].

CUP was once viewed as an almost separate tumor type, with the assumption that its biological properties contribute to the type of presentation, regardless of the site of origin, sometimes with rapid progression and dissemination [2].

## Biological background and proportional distribution according to occult primary site

The biology of CUP is not fully understood and two hypotheses have been put forth. The first one establishes that the tumor can develop without any premalignant lesion or primary tumor. The second one posits that progression is parallel and holds that CUP metastases are an early event in the tumor process [3]. Chromosomal instability was recently suggested as a plausible explanation for CUP’s more aggressive presentations, chemoresistance, as well as their poor prognosis [4]. It has been shown that CUP does not usually display activating point mutations in oncogenes or suppressor genes, but is characterized instead by angiogenesis activation (50–89%), oncogene overexpression (10–30%), hypoxia-related proteins (25%), epithelial–mesenchymal transition markers (16%), and activation of intracellular signals, such as AKT or MAPK (20–35%) [5].

A good quality tissue sample must be obtained to classify the tumor into different histological subtypes to establish a diagnosis of CUP (Tables 1, 2).

**Table 1:** Tumor type and potential occult primary site

Tumor type	%	Potential occult primary (site/types)
Well or moderately differentiated adenocarcinomas	60	Lung, pancreas, hepatobiliary tree, kidney, colon, ovary, breast
Squamous-cell carcinomas	5	Head and neck, lung, cervix, penis, vulva, bladder
Carcinomas with neuroendocrine differentiation	1	Pancreas, GI tract, lung
Poorly differentiated carcinomas (including poorly differentiated adenocarcinomas)	25–30	Adenocarcinoma, melanoma, sarcoma, lymphoma
Undifferentiated neoplasm	5	Carcinoma, lymphoma, germ-cell tumors, melanoma, sarcoma, embryonal carcinoma

**Table 2:** Proportional distribution according to occult primary site

Analysis of 12 postmortem cohort studies (1944–2000), primary tumor site was identified in 644 (73%) out of 884 patients [5]	
Lung 27%	Colorectal 7%
Pancreas 24%	Genital tract 7%
Liver or bile duct 8%	Stomach 6%
Kidney or adrenal 8%	Unknown 27%

## Prognosis

Eighty percent of all patients diagnosed with CUP have poor prognosis and median overall survival of 6 months. A response rate of only 20% and median survival of just 6 months has been reported for these patients when treated with platinum or taxane-based or other combination regimens [6]. Unfavorable subsets include patients with metastatic adenocarcinoma of the liver or other organs, non-papillary malignant ascites (adenocarcinoma), multiple cerebral metastases (adenocarcinoma or squamous carcinoma), several lung or pleural metastases (adenocarcinoma), metastatic lytic bone disease (adenocarcinoma), and squamous-cell carcinoma of the abdominopelvic cavity.

The 20% of CUPs that respond better to therapy and have better prognosis include: men with poorly differentiated carcinoma with midline nodal distribution, squamous-cell carcinoma involving the head and neck lymph nodes, women with papillary adenocarcinoma of the peritoneal cavity or adenocarcinoma affecting only axillary lymph nodes, men with blastic bone metastases and high PSA, neuroendocrine carcinomas of unknown primary site, adenocarcinoma with a colon-cancer profile (CK20+, CK7−, CDX2+), isolated inguinal nodes (squamous carcinoma), and patients with one small, potentially resectable tumor.

CUPs are mainly categorized as having a favorable prognosis or poor-risk. Petrakis et al. separated CUPs into patients with low, intermediate, and high risk by means of a robust multivariate and Classification and Regression Tree (CART) analyses [7]. Predictors of poor patient survival are: male sex, PS > 1, high comorbidity, age older than 64 years, history of smoking (more than 10 pack-years), weight loss, lymphopenia, low serum albumin concentrations, and elevated serum lactate dehydrogenase and alkaline phosphatase concentrations [8].

## Histological diagnosis

The histological confirmation of a malignant metastatic tumor is the cornerstone for CUP; thus, tissue sampling is particularly important. Although cytology or fine-needle biopsy generally provides the initial sample, a core biopsy is recommended for adequate pathological evaluation.

Multidisciplinary collaboration with pathologists and surgeons is crucial at this point to decide on subsequent interventions, such as incisional or excisional biopsy if the sample is inadequate or insufficient to establish diagnosis [1].

After a first evaluation by light microscopy and immunohistochemistry (IHC) staining, CUP can be classified in five morphological subtypes [9]:

1. Well- or moderately differentiated adenocarcinoma (60%),
2. Poorly differentiated adenocarcinoma or undifferentiated carcinoma (29%),
3. squamous-cell carcinoma (5%),
4. poorly differentiated neoplasms (5%), or
5. neuroendocrine tumors (1%).

The pathologist must then exclude tumor types that have specific treatment, such as lymphomas, germ-cell tumors, melanoma, or sarcoma. Further IHC analysis should be performed to aid in identifying the tissue of origin [10, 11].

## Immunohistochemistry tests

**Fig. 1:**
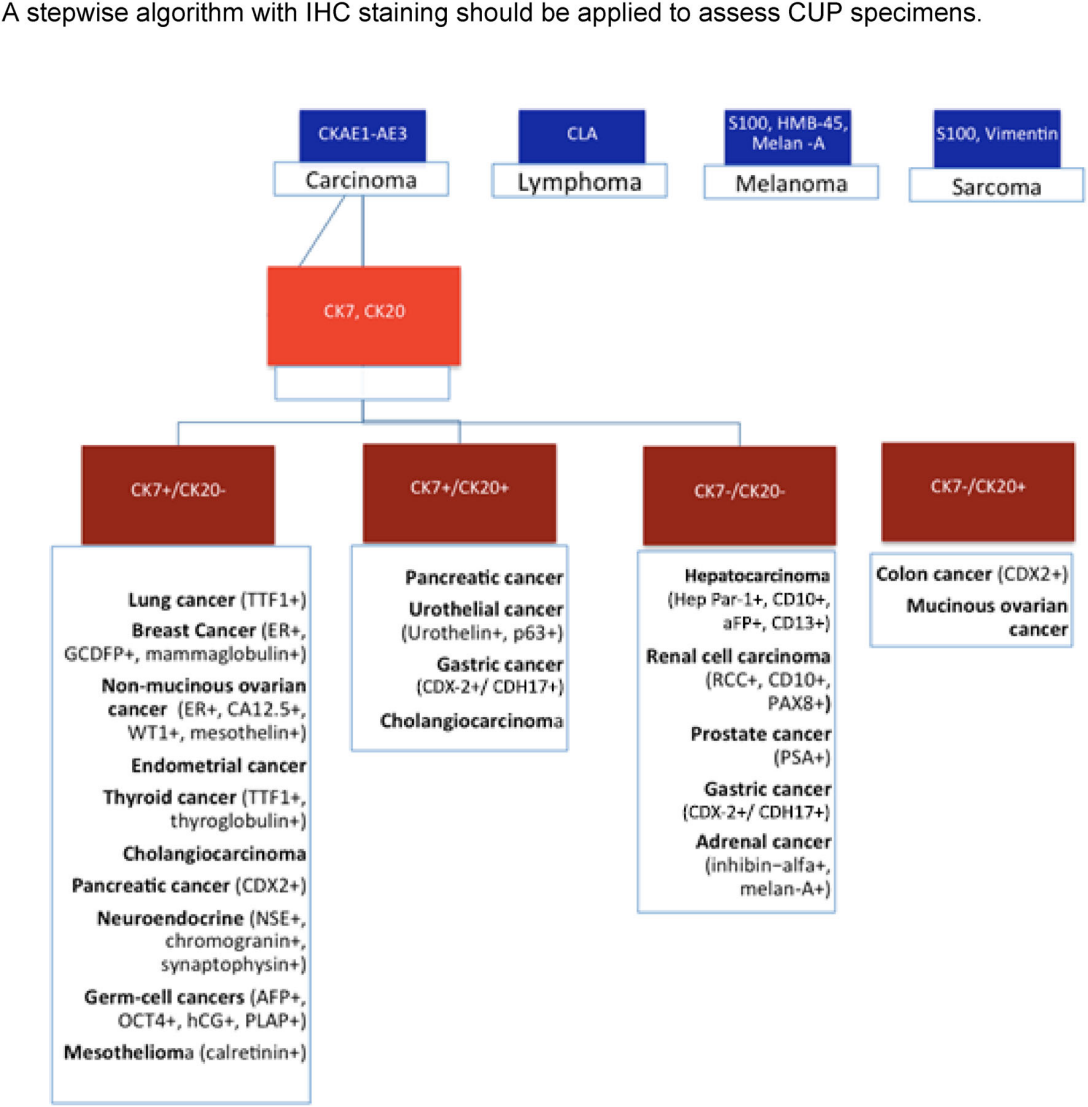
A stepwise algorithm with IHC staining should be applied to assess CUP specimens

IHC testing is cost-effective and should be carried out initially in all CUPs. IHC can provide information about three aspects: the tumor lineage (carcinoma, melanoma, lymphoma, or sarcoma); tumor subtype (adenocarcinoma, germ-cell, hepatocellular, renal, thyroid, neuroendocrine, or squamous-cell cancer), and the primary site of adenocarcinoma (Fig. 1).

IHC staining patterns is capable of identifying the site of origin in < 30% of all CUPs. In patients with poorly differentiated cancers or small biopsy specimens/malignant effusions, IHC staining may not be useful or feasible [12].

The 20 cytokeratin (CK) subtypes are typically expressed in carcinomas. A CK7 plus CK20 staining pattern can point toward additional IHC staining and specific, clinical tests [13]. A CUP having a IHC profile such as CK7+ CK20− TTF1+ suggests lung cancer and bronchoscopy should be performed, whereas CK 7−, CK20+ and CDX2+ suggest colorectal cancer and colonoscopy should be considered.

New IHC markers can provide a more accurate diagnosis, i.e., CDH17 may be a more sensitive marker for gastric cancer than CK20 and CDX2 [14]. Nonetheless, due to tumor heterogeneity, both false positive and false negative IHC staining patterns can be found; for instance, the absence of TTF1 or CDX2 in a minority of lung and colon cancers [15].

Additional IHC markers that characterize melanomas (S100, melan-A, HMB45), sarcomas (vimentin), lymphoma (LCA), neuroendocrin (chromogranin, synaptophysin), prostate (PSA), breast (ER, GCDFP2, mammaglobulin), renal (PAX8), liver (Hepar1, CD13), thyroid (thyroglobulin), or germ-cell (PLAP, OCT4) cancers can be tested when the initial screening is inconclusive.

## Molecular diagnostics

With the application of new knowledge about genetics and molecular biology, and particularly with the development of molecular diagnostic platforms, we find ourselves at the dawn of a revolution in diagnostics, especially of tumors that are difficult to diagnose, as is precisely the case of CUPs.

Given that different cell types have specific patterns of gene expression, the new molecular platforms can make it possible to fine-tune the diagnosis of the possible origin in many cases in which histological techniques are limited. Some of these patterns remain during the process of malignant transformation. Molecular diagnostic platforms present as a useful option to ascertain the primary tumor with a degree of accuracy of 82–97% [16–19] thereby making targeted therapy possible and with it, a better chance for benefit than if non-specific treatment is administered.

There are several molecular platform models; some based on the result of gene expression studies of both mRNA and DNA [16, 17] and others on epigenetics, by identifying the DNA methylation profile [18]. Some molecular platforms have been validated in prospective studies with clinical–pathological criteria, response to medical therapy, identification, identification of primary tumors at autopsy, or on evolution throughout the patient’s life.

In any case, the impact on clinical benefit of targeted treatment based on molecular studies remains controversial and the level of evidence and degree of recommendation is low, due to the fact that they are based on short series, retrospective studies, or phase II studies [18, 19]. Randomized, phase III trials are needed that demonstrate a clear benefit in favor of selecting specific oncological treatments according to molecular study results.

## The diagnostic process

The diagnostic process in patients with CUP seeks to identify subgroups that can benefit from a specific therapeutic procedure, avoiding prolonged, expensive diagnostic processes of scant therapeutic benefit for the patient.

### Anamnesis and physical examination

The first step consists of taking a complete medical history, including toxic habits, medical and surgical history, previous neoplasms or a family history of neoplasms. The physical examination must include head and neck, rectal and testes in males, and pelvic/gynecologic and breasts in women [20].

### Laboratory and radiological examinations

These consist of complete blood count, liver and kidney function tests, electrolytes (including calcium), and lactate dehydrogenase (LDH), since they represent important prognostic factors.

- *Serum tumor markers* PSA should only be determined in males with bone metastases from adenocarcinoma. In patients with metastasis from undifferentiated or poorly differentiated carcinoma in the midline or retroperitoneum, β-HCG and alfaFP should be evaluated to rule out the presence of an extragonadal germ tumor. If hepatocarcinoma is suspected, alfaFP levels must be ascertained. The rest are useful only for monitoring.

Complementary tests to identify the primary in cases of CUP include:

- *Computed tomography* (*CT*) thoraco-abdominal-pelvic CT is customary since, in addition to attempting to detect the primary, it serves as an extension study and can locate lesions that can be biopsied [21].
- *Mammography* should be performed in cases of adenocarcinoma in women.
- *Positron emission tomography* (*PET*) recommended in patients with cervical squamous-cell lymph node involvement, since the primary can be located in one-third of the cases. It is also recommended in those patients who may undergo radical, non-locoregional treatment [22]. Though not otherwise mandatory, a meta-analysis and systematic review of the use of PET in patients with CUP concluded that PET/CT was able to pinpoint the primary tumor in 37% of the cases [23].

#### Examinations to e excluded in the absence of symptoms that indicate otherwise

- Laryngoscopy: useful in cases of cervical lymph node involvement;
- Bronchoscopy: in case of radiological findings such as hilar or mediastinal lymph node involvement, and pulmonary symptoms;
- Gastroscopy: if abdominal symptoms or positive fecal occult blood test;
- Colonoscopy: if abdominal symptoms or positive fecal occult blood test, or biopsy with immunohistochemistry CK20+/CK7−/CDX2+;
- Testicular ultrasound: if retroperitoneal or mediastinal mass;
- Gynecologic ultrasound: if pelvic or peritoneal metastases CK7+ on the biopsy tissue, and
- Breast MRI: if adenocarcinoma with negative mammogram and metastasis to axillary lymph nodes.

## Treatment

Therapy should be individually tailored for each clinical–pathological subset. Between 15 and 20% of CUP are defined as favorable prognostic subsets and should be treated similarly to patients with equivalent known primary site with metastatic dissemination [24] (Table 3).

**Table 3:** Favorable subsets in cancer of unknown primary. Adapted from [24]

Histopathology	Clinical subset	Recommended evaluation^a^	Treatment
Adenocarcinoma	Women with isolated axillary adenopathy	Breast MRI ER/PR/HER-2 stains	Treat as stage II–III breast cancer
	Women with peritoneal carcinomatosis	CA-125	Treat as stage III ovarian cancer
	Men with blastic bone metastases or elevated serum PSA		Treat as metastatic prostate cancer
	Single metastatic site	PET scan	Local therapy ± chemotherapy
Squamous carcinoma	Cervical adenopathy	Endoscopy	Treat as locally advanced
	Inguinal adenopathy	PET scan	Treat as head and neck cancer
			Inguinal node dissection ± radiotherapy ± chemotherapy
Poorly differentiated carcinoma	Young men, mediastinal and/or retroperitoneal mass	HCG, alfaFP	Treat as extragonadal germ-cell tumor
	All others with good performance status	HCG, alfaFP	Treat with empirical CUP regimen

*MRI* magnetic resonance imaging, *ER* estrogen receptor, *PR* progesterone receptor, *PSA* prostate-specific antigen, *HCG* human chorionic gonadotropin, *AFP* alpha-fetoprotein, *CUP* cancer of unknown primary site

^a^In addition to standard evaluation for cancer of unknown primary site

- *Females with peritoneal carcinomatosis* Symptoms of peritoneal carcinomatosis in women should be treated as if it were advanced (stage III–IV) ovarian cancer. Thus, the first therapeutic measure should be exploratory laparotomy with cytoreductive surgery if possible, as it has been proven to increase survival. If resectable, adjuvant chemotherapy should be considered, whereas neoadjuvant treatment with platinum and taxanes may be administered in unresectable cases.
- *Women with axillary nodal metastases* Unilateral axillary nodal adenocarcinoma due to CUP is usually diagnosed in women with an average age of 52 years at diagnosis and its presentation and natural history is similar to breast cancer. More than half of these cases are N2 or N3 and should be treated like breast cancer, with the same indications for neoadjuvant and adjuvant chemotherapy, as well as hormone therapy.
- *Squamous-cell carcinoma with involvement of cervical lymph nodes* These patients should receive trimodal therapy (surgery, chemo- and radiotherapy), as should patients with locally advanced head and neck cancer. In this scenario, PET-FDG with 2-fluoro-2-deoxy-d-glucose is mandatory to detect the primary tumor (25% of cases).
- *Squamous carcinoma involving inguinal lymph nodes* Lymphadenectomy with or without postoperative radiation therapy to the inguinal area. Chemotherapy can also be contemplated for this group of patients.
- *Males with bone metastasis and elevated PSA* In all patients with bone metastases from adenocarcinoma, serum PSA should be quantified. Elevation of this marker should be considered indicative of metastatic prostate cancer and should be treated with hormone therapy and chemotherapy with the same sequence as in prostate cancer.
- *Males with midline nodes* Lymph nodes located in midline structures (more often in the mediastinum) affect young male patients (between 20 and 35 years) and behave similarly to an extragonadal germ-cell tumor. Histologically, it is undifferentiated or poorly differentiated carcinoma. Systemic treatment should be carried out with platinum-based dual agent chemotherapy, which achieves high response rates (overall response, 45–65%; complete response, 20–25%).

Nevertheless, most patients with CUP do not belong to any specific subset and have unfavorable prognoses, despite management with a variety of chemotherapeutic combinations [25]. No specific schedule can be recommended as standard of care but doublets with platinum may be a reasonable choice. A randomized phase III with paclitaxel/carboplatin/etoposid versus gemcitabine/irinotecan in the first line not was associated with a significant improvement in overall survival and median progression-free survival. Triplets do not bring benefit and are more toxic [19, 26, 27]. Modest survival benefits and symptom palliation, as well as preservation of quality of life are the treatment goals in these cases. Consequently, low-toxicity chemotherapy regimens should be administered to poor-risk CUP patients (Table 4).

**Table 4:** Chemotherapy regimens for cancer of unknown primary site

Chemotherapy (mg/m^2^)	Interval (weeks)	Histopathology
Paclitaxel 175 Day 1 Carboplatin 5 AUC Day 1	3	Adenoca and SCC
Docetaxel 75 Day 1 Carboplatin 5 AUC Day 1	3	Adenoca and SCC
Cisplatin 60–75 Day 1 Gemcitabine 1000 Days 1 and 8	3	Adenoca and SCC
Docetaxel 75 Day 1 Gemcitabine 1000 Days 1 and 8	3	Adenoca
Oxaliplatin 85–130 Day 1 Capecitabine 2000 Days 1 and 8	3	Adenoca
Gemcitabine 1000 Days 1 and 8 Irinotecan 100 Days 1 and 8	3	Adenoca
Oxaliplatin 85 Leucovorin 400 5FU 400 bolus 5FU 2400 48 h continuous infusion	2	SCC
Docetaxel 75 Day 1 Cisplatin 75 Day 1 5FU 750 Days 1–5 continuous infusion	3	SCC
Cisplatin 20 Days 1–5 5FU 700 Days 1–5 continuous infusion	3	SCC
Cisplatin 75 Day 1 Etoposide Day 1–3	3	Poorly differentiated carcinoma Neuroendocrine-feature CUP

*Adenoca* adenocarcinoma, *SCC* squamous-cell carcinoma, *CUP* cancer of unknown primary site

Whether or not targeted agents should be used in patients with CUP remains an open question [28]. According to a phase II trial, the combination of bevacizumab and erlotinib, alone or combined with paclitaxel and carboplatin, has been reported to exhibit substantial activity as first or second line treatment with median progression-free survival of 8 months and 27% overall survival at 24 months.

Empirical chemotherapy remains the treatment of choice for patients whose molecular profile is unable to predict tumor origin. We recommend that patients participate in clinical trials whenever possible.

## Surgery or radiotherapy in CUP

Local treatment, such as radical surgery or radiation therapy, should be proposed to patients diagnosed with CUP with a single lesion after complete staging (including PET-CT) [29]. The most common sites are the liver, bone, lung, skin, adrenal gland, and lymph nodes. In most cases, other metastatic locations become evident within a short time, but local treatment can sometimes result in a long disease-free interval.

The first treatment to consider should be tumor resection; however, if the solitary lesion is eligible, definitive radiation should be proposed. In any case, patients with a single metastasis present have a favorable prognosis. Systemic treatment in the neoadjuvant or adjuvant setting continues to be debatable. Nevertheless, empirical adjuvant chemotherapy is reasonable in this setting, particularly in patients with poorly differentiated carcinoma [24].

## Conclusions

CUP accounts for 3–5% of all tumor diagnoses and entails a poor prognosis and median overall survival of 6 months. The limitations currently faced in diagnosing and treating an unknown primary cancer remain a major challenge in comparison with other malignancies.

For the best diagnostic approach, the correct thing is to have enough tumor tissue to be able to carry out the histological and IHC studies. Diagnostic assays have improved significantly over the course of the last decade with the introduction of new IHC stains and when IHC fails to establish an adequate differential diagnosis, molecular tests can help. The new molecular platforms can contribute to fine-tuning the detection of the possible primary in many cases.

The main objective pursued in diagnosing CUP is to identify subgroups that can benefit from a specific treatment procedure, avoiding prolonged, expensive diagnostic processes that offer little therapeutic benefit for the patient.

Therapy should be individualized to suit each clinicopathological subset. Between 15 and 20% of CUP are defined as belonging to favorable prognostic subsets and should be treated similarly to patients with equivalent known primary sites with metastatic dissemination. Empirical chemotherapy remains the treatment of choice for patients whose molecular profile is not able to predict tumor origin. We recommend that patients participate in clinical trials whenever possible.

Randomized clinical trials that compare overall survival and progression-free survival with empirical chemotherapy versus personalized therapy might help to define the standard of care. Still, translational research and molecular diagnostics require further testing. Extending survival or attempts to achieve a cure is possible today only in a subgroup of patients.
